# Predictive Modeling of Microbial Behavior in Food

**DOI:** 10.3390/foods8120654

**Published:** 2019-12-06

**Authors:** E. Stavropoulou, E. Bezirtzoglou

**Affiliations:** 1 Centre Hospitalier Universitaire Vaudois (CHUV), Service de Médecine Interne, CH-1011 Lausanne, Switzerland; 2 Democritus University of Thrace, Medical School, Laboratory of Hygiene and Environmental Protection, Dragana, 68100 Alexandroupolis, Greece

**Keywords:** modeling, predictive microbiology, innovation, food, industry

## Abstract

Microorganisms can contaminate food, thus causing food spoilage and health risks when the food is consumed. Foods are not sterile; they have a natural flora and a transient flora reflecting their environment. To ensure food is safe, we must destroy these microorganisms or prevent their growth. Recurring hazards due to lapses in the handling, processing, and distribution of foods cannot be solved by obsolete methods and inadequate proposals. They require positive approach and resolution through the pooling of accumulated knowledge. As the industrial domain evolves rapidly and we are faced with pressures to continually improve both products and processes, a considerable competitive advantage can be gained by the introduction of predictive modeling in the food industry. Research and development capital concerns of the industry have been preserved by investigating the plethora of factors able to react on the final product. The presence of microorganisms in foods is critical for the quality of the food. However, microbial behavior is closely related to the properties of food itself such as water activity, pH, storage conditions, temperature, and relative humidity. The effect of these factors together contributing to permitting growth of microorganisms in foods can be predicted by mathematical modeling issued from quantitative studies on microbial populations. The use of predictive models permits us to evaluate shifts in microbial numbers in foods from harvesting to production, thus having a permanent and objective evaluation of the involving parameters. In this vein, predictive microbiology is the study of the microbial behavior in relation to certain environmental conditions, which assure food quality and safety. Microbial responses are evaluated through developed mathematical models, which must be validated for the specific case. As a result, predictive microbiology modeling is a useful tool to be applied for quantitative risk assessment. Herein, we review the predictive models that have been adapted for improvement of the food industry chain through a built virtual prototype of the final product or a process reflecting real-world conditions. It is then expected that predictive models are, nowadays, a useful and valuable tool in research as well as in industrial food conservation processes.

## 1. Microorganisms and Food

Microbiology is the scientific discipline that comprises the study of microorganisms (e.g., bacteria, fungi, protozoa, and algae) involved in life cycle chains. It encompasses specialties such as cell biology; genetics; taxonomy; epidemiology; biochemistry; pathogenic bacteriology; food, environmental, industrial, and agricultural microbiology; and microbial ecology. Microbiologists have found microbes living in just about everywhere; soil [1] water [2], air [3], animals [4], plants [5], rocks [6], and humans [7]. Microbes have been around for billions of years because they are able to adapt to the ever-changing environments.

Food microbiology is the study of microorganisms that contaminate food and are involved in foodborne diseases. The foods we consume contain microbes and are rarely sterile. Foods hold microbial loads, the composition of which varies highly. Microorganisms originate from the commensal microflora of raw materials but are also introduced during animal slaughtering and food harvesting, processing, storage, and distribution [8,9]. In most cases, the food is consumed without problematic issues and secondary health effects [9].

According to FAO (Food and Agriculture Organization of the United Nations), more than one-third of all food intended for consumption is wasted and spoiled before it reaches the consumer. This food loss is related to issues during the procedures of harvesting, storage, packaging, and transporting, as well as to national and international institutional and legal structures. Disastrously, the population of undernourished people facing “chronic food deprivation”, increased to 821 million in 2017 compared to 804 million in 2016 (FAO). This indicates a vicious circle as it is strictly associated with the waste of labor places and workers in food industry. Anthropologists used to believe that the carrying capacity of humans on earth without agriculture would be 10 million and this population was reached 10,000 years ago. Agriculture has permitted an important population growth so far by providing an important quantity of food. Nevertheless, during the last few years, the agricultural production has not followed the accelerating population growth and, as a result, the eventual overpopulation of the planet will be followed by mass starvation. In any case, the world’s food supply must boost to keep the balance with population growth, which should be capable of exploiting the land and other resources. With the progress in agriculture, the safe storage of surplus production is of great importance. Microbiological standards have been developed a posteriori to arrest or retard the natural process of food spoilage, and many methods have been developed for this purpose. Food preservation widely depends on decreasing water activity through procedures such as solar drying, salting, concentrated sugar solutions (preserves), or fire-smoking processes [2].

In the 19th century, the development of the food industry progressed with the advancement of the food preservation sector. Industrial procedures such as chilling, canning, and freezing permitted safe importation of foods by remote producers. Currently, the agro-food area is of capital significance for the European and international economies. Food keeps a sovereign role in our life, and safety rules imposed by public authorities and producers are essential. In conclusion, there is a requirement for the implementation of sufficiently easy-to-use and low-cost methods for improving food standards in the aspects of production, storage, and preservation.

As already discussed, foods are never sterile; they carry their permanent microflora and a transitory microflora reflecting their environment [2,7,10]. These microbes are introduced into food from the natural microflora of the raw material or during the procedures of harvesting, slaughtering, and processing [10]. Nevertheless, food is usually consumed without major health consequences ensuing. However, occasionally, microorganisms show their presence by spoiling food or causing foodborne-related diseases in humans [9,10].

It is worth mentioning that some microorganisms are capable of transforming foods beneficially; this is called food fermentation [9,10]. Foods derived after microbial fermentation seem to be beneficial to human health, in particular when lactic acid is present. Lactic acid bacteria have to survive to the stressful conditions of the stomach and the intestine (acid pH and bile). Lactic acid is the major fermented product of a group of bacteria called lactic acid bacteria (LAB).The majority of them have a beneficial impact on the human host by stimulation of the immune system, antiallergic, antimutagenic, hypocholesterolemic effects, and many other [11]. These properties are associated with their probiotic nature. Albeit, it should be noted that characteristics attributed to a probiotic strain are in general strain-specific, and every new strain should be tested for each property.

The most well-known example is *Lactobacillus*, which is involved in the preparation of yoghurt and other dairy products. Live cultures of lactic acid bacteria and *Bifidobacterium* in foods are termed as “probiotics” [12].

Specifically, microorganisms enter the gastrointestinal tract via consumed foods, drinking water, and the breathed-in air [2,4,13]. However, when pathogens are present, infection can occur. Skin lesions, mucosal surfaces, and unwashed hands of food workers are known sources of foodborne diseases. Fecal contamination of foods and waters from humans [7] or animals [3] is another possible source of food contamination, as well as untreated water supplies, flies, and human fingers. In this way, food that has been inadequately cooked, refrigerated, or stored may be heavily infected by microorganisms [9].

Two main categories of food-related diseases are (a) foodborne infections and (b) foodborne intoxications [2,9].

A foodborne infection occurs from the ingestion of pathogens in spoiled food. Following that, the pathogen develops through tissue invasion and/or releasing toxins. If microbial growth happens prior to ingestion, then foodborne intoxication occurs from the consumption of foods containing preformed toxins [10].

Control of *L. monocytogenes* in foods is of great significance due to its ubiquitous nature worldwide in relation with its pathogenic potential. Foodborne listeriosis is a scarce but serious disease including high mortality rates compared with other foodborne microbial pathogenic contaminants.

*L. monocytogenes* is an opportunistic pathogen and the occurring disease basically concerns susceptible layers of population [14]. The European Regulation (EC) [15] No 2073/2005 as reviewed by the Regulation (EC) [16] No 1441/2007, does not establish the limits for *L. monocytogenes* in foods (Buchanan et al., 2017) [17]. It is then conceivable that introduction of predictive models will be an important tool in limiting food spoilage [18,19,20].

*Clostridium botulinum* is isolated from terrestrial, marine, and freshwater environments.

It produces neurotoxins that are known as one of the most potent bacterial toxins. These toxins are not disrupted by digestive enzymes causing foodborne disease [21].

Technical reports all over the world focus on *Campylobacter* as a food contaminant [22,23]. Its primary route of infection being through poultry meat [24]. There is information about introduction of predictive modeling in *Campylobacter* food spoilage [25,26]. Recently, a model was developed, which permits the determination of emerging *Campylobacter* strains within a flock. Additionally, the model selects for phenotypic advantages in order to promptly eliminate demographically weaker strains [27].

Major causes in foods poisoning include *Salmonella spp.* and the serotype *Esherichia coli O157:H7* [28,29]. *E. coli* O157:H7 is causing a serious foodborne disease through consumption of contaminated and raw or undercooked food (raw milk, ground beef).

The disease may lead to hemorrhagic diarrhea and kidney failure.

Salmonella is a leading cause of foodborne illness with an estimated 80.3 million cases worldwide globally per year and 1.4 million cases reported cases in the United States [30]. The main route of infection for Salmonellosis being poultry and eggs. World Health Organization [31] states that more than 23 million per year people are infected by food contaminated Salmonella. Due to its importance for public health, multiple predictive models were proposed to inactivate the microorganism [32].

## 2. Food Control Authorities

Systematic control and inspection of food is imposed in order to assure that consumed food will be healthful and of the quality claimed. Multiple control agencies ranging from international to national, and private as well, are aiming to achieve this goal.

As stated previously, FAO (Food and Agriculture Organization), a branch of United Nations (UN), together with other sections of the UN like the World Health Organization (WHO) and the International Children’s Emergency Fund (UNICEF), without being major enforcement or control agencies, have developed a common attention in healthful and safe food. However, FAO is chiefly focused on food production and safety. In this wavelength, the commission of FAO/WHO Food Standards has been created which established international standards for the food industry among different countries. These standards are published in *Codex Alimentarius.*

The microbiological criteria are defined by the International Commission on Microbiological Specifications for Foods (ICMSF) [33]: (a) The microbiological standard; (b) the microbiological specification, which is applied in the food trade as a condition of acceptance by the consumer; and (c) the microbiological guideline, which is applied in the monitoring of the microbiological acceptability of a product or process. Moreover, the ICMSF also specifies the following criteria [34]: (a) A register of the food to which the criterion supplies; (b) a register of the microorganisms or their involved toxins; (c) a description of the qualitative and quantitative methodologies applied for detection of the microorganisms and their toxins; (d) the numbers and sizes of samples to be taken; and (e) the microbiological cut-off for the given product as well as the number of repeated samples in order to qualify a product as being acceptable.

The Food and Drug Administration (FDA) of the Department of Health, Education, and Welfare (HEW) in USA ensures food safety as well as the correct labelling of the involved food. Moreover, in the USA, the United States Department of Agriculture (USDA) has legislative authority to promote safe agricultural products such as meat, eggs, and poultry without forgetting the National Marine Fishery Service (NMFS) concerned with the inspection of fisheries products [34].

In Europe, the creation of an autonomous European Food Authority has ensured a high level of food safety. This authority has a role in providing scientific advice on various food domains as well as quick effective operations and measures to ensure consumers’ health. Moreover, the authority is in close contact with all national and international scientific bodies. The European Food Authority provides the European Commission with the necessary data to inform actions that should be taken (http://www.efsa.europa.eu/).

Following the European Commission’s paper on food law (COM (97)176 final); European Commission, 1997 [35], a new legal framework was suggested. It covers the levels of the food chain, together with issues of animal feeding production, to ensure effective protection of consumers’ health. It imposes rules and actions for the safe industrial production of foods [36]. It is worthy of note that the systematic control of contaminants and residues in foodstuffs is a substantial action for securing consumer protection in the European Union, as residues of animal or vegetal origin may constitute intrinsic hazards. In this vein, in order to protect the consumer from the different risks in food products, the legislative framework lays out multiple hygienic measures based on hazard analysis and critical control point (HACCP) principles, principally under the name of “microbiological criteria” [37].

Microbiological criteria are tools that can be used in assessing the safety and quality of foods.

In this vein, in every country, there are developed national organizations to establish quality standards to be met by foods provided for consumption and assure food safety.

Predictive models illustrating microbial behavior in foods are an asset for industry, food safety authorities, and education policies. Industry can use such models to shape food and to implement food safety in its management, processing, distribution, and storage. Predictive modeling is important during food manufacturing operations. It helps to design factory heating processes, establish critical control points (CCPs) in HACCP, assessment of the impact of procedural deviations on microbiological safety, and food quality. Moreover, it permits to estimate the impact on final product quality and consumer safety.

Food and health safety authorities can perform risk assessment and develop the food safety regulations on predictions. Moreover, education can apply predictive modeling to get knowledge in scientists for a better understanding the different processing technologies act on microorganisms and their behavior. Without any doubt, industries and institutions using microbiological models are refining the time-consuming and expensive process of classical microbial testing by developing specific databases.

## 3. Food Preservation

The industrial setting in which food processing is waged is an important factor for the quality of the product [2,9,34]. The main objective should be the design of hygienic food-processing equipment. Cleaning procedures are of capital importance as well. Despite the application of thorough hygienic techniques, germs are not completely eliminated from foods by these cleaning procedures solely, and the introduction of more effective approaches is required to preserve the food quality.

A non-profit group was established in 1989, the European Hygienic Engineering and Design Group (EHEDG) from equipment manufacturers, food producers, suppliers to the food industry, and also public health and governmental authorities as well as Universities and Research Centers involved in the field. EHEDG aims to the improvement of the engineering hygienic conditions in food plants by adapting legislation in order to produce safe food. European directives must be respected under guidance of EHEDG in items of food hygiene, food contact materials, and machinery (EC Directive 2006/42/EC for Machinery, EN 1672-2 and EN ISO 14159 on Hygiene requirements for the design of machinery) in conformity with international and national legislation and directives.

In the following sections, we review some of the methods used for controlling the microbiological quality of foods. Investment toward mathematic modeling in the food industry [11] for quantifying the microbial inactivation due to an imposed environmental stress [13] or preservation barriers is discussed extensively through this review in order to provide an innovative and sustainable profile for the food industry domain. Firstly, based on the control of the microbiological quality of foods, specific criteria must be adapted for exploring the term “quality”, which defines the degree of excellence possessed by a product [2] frequently and rigorously.

During the Paleolithic era, we settled on the earth, man sought his food, and for this purpose, the mass movements of the population were made in order to find more fertile locations that would provide his food. Since that era, human population grew and the need for food consumption increased, which was followed by excess food production. The requirement of food preservation and safe food production became important. Empirical and traditional methods were developed to limit food spoilage. Those methods included cooking, smoking over a fire, salting, addition of spices, and others.

In our industrialized society, food is preserved by a variety of physicochemical methods including thermal treatment, pasteurization, canning, irradiation, filtration, freeze-drying, vacuum packing, and addition of different preservatives.

The hurdle effect is a combination of different barriers for foods that touch up the production of a qualified and hygienic product. Hurdles are physicochemical parameters responsible for ensuring a safety and stable food. There are mentioned more than 60 potential hurdles to be associated with food. However, the most commonly studied hurdles as a preservation strategy are focused on controlling temperature, water activity, redox potential, and acidity.

In this vein, efforts have been done to provide a qualitative assessment of the limits in order to ensure the safety of food. Thus, the conception of developing the means to implement predictive modeling to hurdle technology seems to be of high interest for the industrial world.

Nonetheless, it is not clarified as to what extent hurdle parameters cooperate influencing growth of microorganisms in foods.

In an attempt to bring to light the concept of predictive microbiology and stimulate the interest of the scientific and industrial world, we hereby tried to cover several basic aspects of field.

## 4. Development of Modeling Systems in the Food Industry

The setting of maximal or minimal permitting levels of tolerances is an essential part of the enforcement structure. Such levels must be set, however, with full and realistic approaches by the proposed mathematic models [38]. Moreover, as experimental techniques are improved and adapted, modeling will provide us with a detailed account of occurring or upcoming problems that can disrupt the existing production chain [39].

As stated already, all involved private and public authorities could perform risk analysis and develop the food safety standards, regulations, and predictions.

Systematic and vigorous modeling applications must be initiated before effective regulation of the problem can be established. This implies that there must be some effective methods for predicting behavior of a food during its production, considered together with the environmental conditions involved [40].

In terms of microbiology, predictions must be implemented by determining the qualitative and quantitative development of the microflora. The HACCP system [41] proposed and applied during recent years in industries consists of a systematic and continuous detection of safety points in the production chain and overseeing monitoring methods.

The term “quantitative microbial ecology” has been suggested [42] for the modeling studies of colonizing microflora. However, an alternative to this term, “predictive microbiology,” seems to be applied nowadays as it brought solutions to the above-formulated problems by modeling the microflora’s evolution, thus allowing the earlier prediction and information about product procedures and quality by similar means [40,43,44].

Modeling [45,46] essentially represents the relationships between the inputs and outputs of a given system. The thermal destruction of bacterial species in the microflora achieved from the information obtained after modeling of the rate of a chemical reaction or the temperature is striking evidence of the approach’s effectiveness [47]. Objective methods of measurement (e.g., for chemical substance, microbiological, and sensorial analyses) are necessary to detect the factors that limit the product’s life span. The product life span [48] is the period of adequate storage of a product before consumption, as there is a substantial risk of the product becoming dangerous or developing organoleptic spoilage beyond this date. This period is slightly more extensive than the product shelf-life. In fact, the prediction could apply to different conditions [49]; that is, prediction of the product’s life span from the design stage, or prediction of the total product life span for a product aliquot coming out of the production chain.

Mathematical models allow the prediction of microbial behavior, which affects its growth under different environment conditions. These conditions are basically grouped in two main categories; intrinsic and extrinsic factors. Intrinsic factors comprise the physicochemical properties of the food itself like pH, water activity, and redox potential. Extrinsic factors are noted as all environmental factors responsible for the limiting of the microbial growth, such as temperature, relative humidity, and gaseous atmosphere.

## 5. Mathematical Models for Predictive Microbiology

*Kinetic models* [50] can predict the concentration levels attached to a given microbial strain, and thus the onset of the upcoming risk is determined (infection or intoxication-associated risk). These models are calculated with the rates of growth or death response.

*Probability models* have been used to predict production of toxins by microorganisms [51,52]. Their use was broadened lately in defined environments p.e. under stress [53]. These models only suggest the probability of bacterial growth and their toxins but not the speed with which the effect occurs.

The grouping of two main types of models has been effective: *Empirical and mechanistic models* [44,54].

*Empirical models* offer mathematical relationships between inputs and outputs without any linkage of the structure to a physicochemical or other parameter. They allow us to relate two variables through a polynomial equation. The main disadvantage of such models is their inability to validate concrete developed conditions. Nonetheless, this obviously simple procedure is in fact offered by spreadsheet software equipped with graph-plotting schedules. For example, empirical models based on two-variable relationships allow us to correlate the final product quality attained with the procedure time.

*Mechanistic models* are more flexible, allowing us to determine the different parameters. For their development, a good extensive knowledge of the field to be applied is required. Mechanistic models are usually used as an index of prediction when conditions are modified. More specifically, they permit us to determine whether a developed model theory is effective for the predicted experimental conditions and challenges, and thereafter to test the model by experimentation [39,55]. Besides this, we must note that mechanistic models cannot be extrapolated to situations outside the range from which they derive, or they rarely apply to that within a given degree of confidence.

Hereby, we grouped on a table the most common predictive mathematical models in order to offer a better understanding of their use (Table 1).

Subsequently, we will describe the different types of modeling techniques applied in the food industry [57,58]. As already discussed, modeling the effect of temperature on microbial growth and behavior is well known and has been extensively studied [42,44,53,56].

Basically, the *Arrhenius model* was proposed according to the following equation [11,44,53,57,58,59].

*r* = *A*.*e* − μ/*RT*

where *r* is the growth rate, μ is the activation energy in cal/mol, *R* is the universal gas constant, *A* is a constant, and *T* is the storage temperature in degrees Kelvin.

Frequently, when microbes enter a new environment, they do not begin to multiply immediately but require a period of adjustment to the new environment before they begin to increase their numbers by cell division. In this phase, which is called the lag phase, the microbial cells repair lesions resulting from earlier injuries or stress [2,7], and old or dormant cells also restore essential constituents that have become depleted or damaged. Prediction of the food storage life span includes a determination of the length of this lag phase, in addition to the obtained growth rate. In this phase, the young microbes synthesize the enzymes necessary to adjust to their new environment. Bacterial cells transferred from a nutritionally rich environment to one that is nutritionally poor also need time to synthesize their enzymes. The duration of the lag phase can be variable depending on the adjustment of microbes to their new environment. In cases where multiplying microbes are inoculated into the same type of culture medium, this phase can be completely absent.

It is then conceivable that the evaluation of two different parameters must be considered from the knowledge obtained of the conditions described above; namely, the specific growth rate, which is dependent on the nature of the medium and the incubation temperature, and the length of the lag phase, which is dependent on the stress exposure conditions. The Arrhenius equation is then applied for determining the kinetic bacterial growth curve at a given temperature. It also allows us to determine the kinetic bacterial growth curves at other temperatures to predict the levels reached after a determined storage time. The Arrhenius model is applied not only in a satisfactory way for the modeling of microbiological processes, but is also suitable for predicting chemical spoilage processes. In this latter case, it is represented graphically by a line of slope -*K*2, despite that the relation in a microbial growth curve is not linear [11,42,44].

To explain this last fact, Schoolfield [60] developed a nonlinear regression equation derived from the Arrhenius equation above, describing the effect of temperature on biological ecosystems. This consisted of a complex expression and could give us important information about such facts as the conditions of milk pasteurization, in which the limiting factor of the milk life span is considered to be bacterial growth. The model gives the time needed for a 1000-fold bacterial multiplication at different combinations of temperature, pH, and water activity.

Another model was later proposed based on empirical concepts by Ratowsky [61]. Their model reports the relationship between the growth rate and temperature as being linear, at least for the range between the minimum and optimum growth temperatures. However, recent specific applications of the model have suggested that the Schoolfield model is more reliable, especially at low temperatures [60]. Although the knowledge obtained from the above models is still limited, their derivatives seem to be quite satisfactory and pertinent.

Since then, different types of modeling techniques have been developed (Table 2) [62,63]. *Multi-factorial models* [43,50] predict the growth of microbial populations by analyzing the contribution of essential factors involved in the system. *Bi-dimensional and tridimensional models* combine the different factors incriminated in the food chain [63]. *Response surface methodology* [42] is a common model used in the field of optimization. This model is adaptable when the processing conditions or other factors vary systematically. It permits the measurement of the output of a system that is dependent on a number of input variables and the development of experimental designs. The relationship is not linear, as each variable is dependent on its own complex equation.

The previously described models are integrated in easy to use software applications.

Software packages are available to facilitate the modeling methodology. Those tools are called *tertiary models* and are extensively used in food industries.

The ComBase predictive models software is another tertiary online model tool based on ComBase data to predict the growth or inactivation of microorganisms for studies of quantitative food microbiology. The tool was developed by the University of Tasmania and the USDA Agricultural Research Service (USDA-ARS) [64]. It includes an important database of more than 60,000 records deposited into ComBase. Mathematical models (ComBase models) have developed on constantly recorded data to predict how microorganisms behave under environmental conditions (https://www.combase.cc/index.php/en/).

Another known tertiary predictive model is the pathogen modeling program (PMP, v.7.0), which was developed by the USDA-ARS Agricultural Research Service [65]. The PMP (pathogen modeling program) [65] calculates the pathogens growth, survival, or inactivation as a function of different factors and conditions, such as temperature, pH, sodium pyrophosphate, and sodium chloride concentration, by the use of a straightforward user interface [66]. This model is of high interest for evaluating the potential risk associated with microbiological hazards.

In this purpose, two types of models have been proposed: Stand-alone and excel models. The proposed models are constantly upgraded and new models are developed.

The growth predictor (GP), provided from the United Kingdom, is a download package concerned with microbial growth. The model is available at www.ifr.ac.uk/safety/growth Predictor [67].

The DMfit Program uses Baranyi and Roberts, 1995 [55] model to fit curves with growth data by linear and nonlinear regression.

Other predictive microbiology tools are reported by the UK Institute of Food Research and are available at http://www.combase.cc/tools/.

Other specific tertiary models are available. The model seafood spoilage predictor (SSP) focuses on spoilage of fresh fish [68,69], while the food spoilage predictor (FSP) predicts the food pathogen *Pseudomonas* [70], available at http://www.hdl.com.au/html.body_fsp.htm.

Another model for predicting shelf life, safety, and quality of ready-to-eat food products is the SOPHY (software tool for prediction of ready-to-eat food product shelf life, quality, and safety) which is available at: https://dev2.chainfood.com [71].

The GroPIN model was developed by a Greek team (Psomas and Skandamis, [72]). This tertiary model includes 367 published models concerning 29 pathogenic and 43 spoilage organisms in multiple foods of vegetal or animal origin. The model is available at the website: http://www.aua.gr/psomas/gropin/ [73].

Without any doubt, multiple predictive microbiology software tools have been developed during the last years. Recently, at the 8th International Conference on Predictive Modelling in Food, which was held in Paris, an extensive discussion on their utility was stated [74].

Specific models have been proposed to explain the relationship between the force applied and the extension of a spring (*Hooke’s law*) [42]. *Computational fluid dynamics* [42] calculates the motion of a fluid or mixing process in given equipment and explains the rate of change imposed after defection or perturbation of the given system. Through use of the finite increment method by a process of integration, the rate equation can be converted into a concentration–time or distance-time relationship. The rate of change must be successively calculated, and this is, in fact, easily executed by applying modern computer programs.

*Probabilistic models* [42,48] can predict the probability of a microbial response under certain conditions (i.e., probability of toxin production or strain sporulation). This model is based on the condition that a certain structure is formed from multiple random events, starting on a molecular level. We can then assume that the average of a very large number of particles (the bulk) of a given system can be predicted. The distribution of additive intakes in food can be formulated by probabilistic modeling. Constructively, the most significant parameter associated with a product’s life span is the microbial population growing in it. Thus, it is obvious that modeling must include a relationship of the microbial population with physicochemical factors, such as the temperature, pH, water activity, and substrate or inhibitor concentration. More recently, a model without a theoretical basis was developed that included parameters of clear biological significance; it might be considered as a Taylor series approximation of the theoretical polynomial function [48].

## 6. Classification Models for Predictive Microbiology

Considering the above, a three-level classification scheme has been proposed as primary, secondary, and tertiary models for measuring the bacterial behavior [75].

*Primary models* measure the behavior of the bacteria over time to a set of conditions. They include bacterial growth models [76,77], bacterial death models [78], growth rate models [42], thermal inactivation models [79], and others.

The most common to fit the microbial growth data seem to be the sigmoidal functions. The sigmoidal functions are composed of four distinct phases as is the case of the microbial growth curve. Two models proposed by Gibson et al. (1997) [76], the *modified logistic model* and the *modified Gompertz,* are broadly used.

In this purpose, primary models use the curve-fitting tool of Matlab 7.0 (Math Works, Natick, MA, USA) with which 95% confidence limit (CL) for growth parameters is usually applied. Hence, it is believed that a considerable part of microbial population under the same environmental conditions present similar growth potential. However, when modelling growth curves are obtained by different methodologies (p.e. colony forming units counting or optical density), the fitted parameters show differentiations, as the rate of increase of the optical absorbance does not utter as the maximum specific growth rate and the detection time is not equal to the lag time; furthermore, the initial inoculum is much higher than the detection threshold. Recently, new techniques were developed on processing microscopic procedures issued from monitoring bacterial colony growth. The microscopic images are collected and related to bacterial growth [80].

From image processing, information regarding, e.g., morphology, the colony radius and colony area is gathered and related to bacterial growth. 

Nevertheless, the *Baranyi model* proposed in the 1990s [59] has been extensively investigated and used for modeling purposes of the microbial growth. Moreover, by the use of the curve-fitting tool programs DMFit, an Excel add-in, and MicroFit, a stand-alone fitting program, which is allocated by the Institute of Food Research in the U.K. (http://www.ifr.bbsrc.ac.uk/Safety/DMFit/default.html), its use has become widely known.

As far as the *model of Buchanan* is concerned, which is a three-phase linear model (lag phase; exponential growth phase; and stationary phase), its use seems to be limited. The model was used to fit experimental data for *E. coli* O157:H7.

Albeit, the above models are fitting results in case of homogenous populations. *McKellar* proposes a model in case that growth is expressed as a function of two distinct cell populations.

Lastly, the *Gamma concept model* assumes that the effects of controlling variables can be broadening and that the cardinal parameters of temperature, pH, and water activity are not dependent on the other variables [54].

The *secondary models* control factors of primary models changing the kinetic parameters (p.e. modeling of lag phase and growth rate with respect to one or more environmental or physicochemical factors [54,61]. In other terms, we can say that secondary models characterize all those biotic and abiotic parameters able to modify the microbial kinetics, such as temperature, water activity, pH, and other factors [81].

Finally, the *tertiary models* are applications of one or more secondary models for providing predictions by including algorithms to calculate shifting conditions. These models are computer tools to consolidate the primary and secondary models used broadly in the food industry and research [82].

## 7. Model Validation

Data gathered for exploitation in modeling must be issued from a uniform environment, based on the knowledge that the bacteriological media selected give different bacterial growth results due to the limitations of the methods and media used. Other parameters affecting the acquired data must also be taken into consideration following the presented case. Finally, after development, the model must be validated by comparing the values it produces with a sufficient number of experiments.

The food industry is vital in terms of profitability, investment, worldwide exports, and workforce employment. A common element for improving the capacity of the food industry is the requirement of improved conditions and workforce skills. Predictive microbiology generally focuses on the potential overgrowth of spoilage bacteria and foodborne pathogens in foods. As mentioned above, some bacteria act in a beneficial way and are applied in food fermentation processes. Recently, the attention of scientists has been focused on “beneficial microorganisms.” Predictive modeling helps to gain a systematic understanding of the influence of environmental conditions (temperature, pH, salt, etc.) that should prevail in food fermentation processes, by determining the functionality of novel starter cultures in simulation media [83]. A quantitative predictive model has been developed to account for both the adherence of microorganisms to surfaces and their conditions of logarithmic growth in food chains [57]. The model permits researchers to shape the optimal processing conditions and procedures in order to limit bacterial contamination, and to decide on the critical points with regard to the food hygiene status.

Thus, a modeling approach clarifies the close relationship between foods and bacterial growth. Furthermore, since bacterial growth is often dependent on the nature of the food involved, the development of mathematical models may be helpful for predicting the behavior of the bacterial population in the food matrix, hence, to quantify solutions and ensure a safe food supply.

## 8. Applications of Models

In this section, we report examples of the use of several mathematical models that have been successfully used and proposed for application.

In an attempt to evaluate the kinetics of *L. monocytogenes* in fishery products, measurements of optical density (OD) under different atmosphere conditions such as reduced oxygen and aerobic environment were realized. The Baranyi model has been applied to evaluate the maximum growth rate (*μ*_max_) from the obtained growth curves. Moreover, the effect of storage temperature on *μ*_max_ was estimated by modeling using the Ratkowsky square root model. All developed models were validated. Safe predictions were provided for *L. monocytogenes* in the fishery products by the developed models [84].

Another team [45] described a model for the microbial interaction and the death of *Escherichia coli* O157:H7 during the fermentation of green table olives. For this purpose, two different starter cultures and various amounts of glucose and sucrose were used. During fermentation, high amounts of lactic acid were produced under these stressful conditions. In this context, the death of *E. Coli* O157:H7 was evaluated by a differential equation including multiple factors such as pH, protective effect of the substrate, and protonated lactic acid.

Four primary models were used by other authors [85] for studying the growth of the yeast *Pichia anomala* in olive fermentation, as follows; the modified Gompertz, modified logistic, modified Richards-Stannard, and finally, the Baranyi-Roberts model. Hence, the maximum specific growth rate (mmax) and lag phase period from the growth curves were determined. In spite of the good fit of all models, the modified Gompertz and Richards–Stannard models were shown to be the most suitable.

However, other authors, by studying the inactivation of *Salmonella enterica* serotype *Agona,* concluded that kinetics models are valid only for large populations, as in small populations, the D-value presents a high variability due to the cell heterogeneity of the population. The authors proposed characterization by a probability distribution in order to quantify the variability in the inactivation of mixed microbial populations [86].

The influence of the pulsed light technology (PL) on the kinetics of *Bacillus cereus* spores surviving the treatment was studied [87]. PL seems to react on the kinetic parameters of the microorganism. The *μ*_max_ decreased with increasing intensity. A polynomial regression was adjusted between the *μ*_max_ of the survivors and the final inactivation. As a result, PL treated foods would have longer keeping capacities (shelf-life) than those treated by other thermal or irradiation procedures.

## 9. Conclusions

As the control of products from supply areas to processing plants and then on to the markets is a critical factor for efficiency and food quality, extensive research is required, including the development of an easily accessible database of reliable information on the microbial responses to food-processing conditions. Because of the fact that numerous conditions intervene in the food supply chain, such as industrial qualification and risks relevant to food production, it is obvious that the variety of structures and processes permitting technological and structural shifts in the industry must be assessed. We must assume that the contamination pathway in the food chain holds a capital role for the preservation of the industrial production of foods. Notwithstanding this, the majority of scientists involved in the food industry seem unaware of the immense potential and accessibility of the available modeling tools The value of model creation as well as for the implementation of more research should be understood from scientific community and a multiscientific team should be formed consisting of mathematicians, chemists, and biologists for defining which factors could be of the highest risk for a product’s quality. Through this paper, we have evaluated predictive models and other novel food industry-associated approaches and support tools that could be invaluable for seeking definite solutions to specific problems related to the efficiency of the food chain industry.

## Figures and Tables

**Table 1:** Kind of mathematical models applied in food industry.

Model Type	Publication	Prediction
Kinetic	(Smith and Schaffner, 2004) [50]	Rate of growth or death response (concentration level of microbial strain) Chemical spoilage prediction
Probabilistic	(Stumbo et al., 1983) [51]; (Fakruddin, 2011) [52]; (Baker and Genigeorgis, 1990) [53]	Production of toxins by microorganisms or sporulation
Empirical	(Buchanan, 1990) [54]; (Baranyi and Roberts, 1995) [55] (Wedzicha and Roberts, 2006) [45]	Relationships between inputs and outputs Two variables relation through a polynomial equation
Mechanistic	(Gaucher, 2003) [56] (Valdramidis, 2006) [39]	Index of prediction under modified conditions, Determination of different parameters

**Table 2:** Different types of modeling techniques.

Model Type	Publication	Prediction
**Multi-factorial models**	(Bourgeois and Leveau, 1995; McMeekin et al., 1997) [42,48]	Growth of microbial population considering involved factors
**Bi-dimensional and tridimensional models**	(Liu et al., 2015) [63] (Baranyi and Buss da Siva., 2017) [59]	Growth of microbial population by combination of multiple involved factors
**Response surface methodology**	(McMeekin et al., 1997) [42]	Measurement of the system output dependent on input variables, when factors and conditions vary systematically
**ComBase models**	University of Tasmania and the USDA Agricultural Research Service (USDA-ARS) [64]	Behavior of microorganisms under environmental conditions
**USDA-ARS Agricultural Research Service**	USDA,2003 (PMP, v.7.0) (Marks, 2008) [65]	Growth of pathogens, survival, or inactivation as a function of different factors
**Growth predictor (GP)**	UK (Quadram Institute Bioscience)	Growth of microbial population
**DMfit Program**	UK Institute for Food Research	Comparison of the specific growth rates of different bacterial growth curves and statistical significance
**Seafood Spoilage Predictor (SSP)**	(Dalgaard, 1995; Gram and Dalgaard, 2002) [68,69]	Prediction of fresh fish spoilage
**Food Spoilage Predictor (FSP)**	(Neumeyer et al. 1997) [70]	Prediction of the food pathogen *Pseudomonas*
**SOPHY (SOftware tool for Prediction of ready-to-eat food product sHelf life, quality and safetY)**	BREMERHAVEN EV-Germany	Prediction of shelf life, safety, and quality of ready-to-eat foods
**Computational fluid dynamics**	(McMeekin et al., 1997) [42]	Fluid dynamics after perturbation or defection of the system The model is applied for liquid foods
**GroPIN model**	(Psomas and Skandamis, Agricultural university of Athens, Greece, 2019 update) [72]	Growth of pathogens and spoilage microorganism as a function of different intrinsic and extrinsic factors
